# Pathogen-Specific T Cell Polyfunctionality Is a Correlate of T Cell Efficacy and Immune Protection

**DOI:** 10.1371/journal.pone.0128714

**Published:** 2015-06-05

**Authors:** Anders Boyd, Jorge R. Almeida, Patricia A. Darrah, Delphine Sauce, Robert A. Seder, Victor Appay, Guy Gorochov, Martin Larsen

**Affiliations:** 1 Inserm UMR-S1136, Institut Pierre Louis d’Epidémiologie et de Santé Publique, Paris, France; 2 Inserm UMR-S1135, Centre d’Immunologie et des Maladies Infectieuses (CIMI-Paris), Paris, France; 3 Vaccine Research Center (VRC), National Institute of Allergy and Infectious Diseases, National Institutes of Health (NIH), Bethesda, MD, United States of America; 4 AP-HP, Groupement Hospitalier Pitié-Salpêtrière, Département d’Immunologie, Paris, France; 5 Sorbonne Universités, UPMC Univ Paris 06, CR7, CIMI-Paris, Paris, France; INRS - Institut Armand Frappier, CANADA

## Abstract

**Introduction:**

Understanding the factors that delineate the efficacy of T cell responses towards pathogens is crucial for our ability to develop potent therapies against infectious diseases. Multidimensional evaluation of T cell functionality at the single-cell level enables exhaustive analysis of combinatorial functional properties, hence polyfunctionality. We have recently invented an algorithm that quantifies polyfunctionality, the Polyfunctionality Index (Larsen *et al*. PLoS One 2012). Here we demonstrate that quantitative assessment of T cell polyfunctionality correlates with T cell efficacy measured as the capacity to kill target cells *in vitro* and control infection *in vivo*.

**Methods:**

We employed the polyfunctionality index on two datasets selected for their unique ability to evaluate the polyfunctional imprint on T cell efficacy. 1) HIV-specific CD8^+^ T cells and 2) *Leishmania major*-specific CD4^+^ T cells were analysed for their capacity to secrete multiple effector molecules, kill target cells and control infection. Briefly, employing the Polyfunctionality Index algorithm we determined the parameter estimates resulting in optimal correlation between T cell polyfunctionality and T cell efficacy.

**Results:**

T cell polyfunctionality is correlated with T cell efficacy measured as 1) target killing (r=0.807, P<0.0001) and 2) lesion size upon challenge with *Leishmania major* (r=-0.50, P=0.004). Contrary to an approach relying on the Polyfunctionality Index algorithm, quantitative evaluation of T cell polyfunctionality traditionally ignores the gradual contribution of more or less polyfunctional T cells. Indeed, comparing both approaches we show that optimal description of T cell efficacy is obtained when gradually integrating all levels of polyfunctionality in accordance with the Polyfunctionality Index.

**Conclusions:**

Our study presents a generalizable methodology to objectively evaluate the impact of polyfunctionality on T cell efficacy. We show that T cell polyfunctionality is a superior correlate of T cell efficacy both *in vitro* and *in vivo* as compared with response size. Therefore, future immunotherapies should aim to increase T cell polyfunctionality.

## Introduction

Pathogens compose a major socio-economic challenge to modern society. Humans are able to develop pathogen-specific immunity, which is induced either naturally (pathogen infection) or artificially (vaccination). Such immunity is supposed to confer protection by 1) antibody mediated neutralisation and elimination of pathogens, or to control infection through 2) T cell mediated elimination of infected host cells. Understanding the factors that delineate the efficacy of antibody and T cell responses towards pathogens is crucial for our ability to develop potent therapies.

T cells play important roles in the series of highly coordinated immune events that lead to pathogen clearance. Indeed, they are directly involved in the eradication of infected host cells, but they are also inherently communicating with innate immunity and pathogen-specific antibody development, which are crucial for pathogen clearance. It is custom to analyse the effect of T cells at different levels, 1) pathogen clearance and clinical recovery, 2) target killing, cellular help and recruitment of innate immune cells and 3) effector molecules expressed by T cells. Whereas T cell efficacy is typically evaluated extrinsically (level 1 and 2), their functionality is more often analysed intrinsically (level 3). Indeed, T cell functionality assays have the advantage of being applicable to large cohorts as well as many cell types and subsets in a standardized manner, with readouts that can be highly multiparametric. Here, we focus on how to associate or even predict extrinsic T cell efficacy from intrinsic T cell functionality. Using highly multiparametric datasets of T cell polyfunctionality we also propose a widely applicable analytical strategy, which objectively identifies the importance of individual and combinatorial effector functions.

Functional evaluation of T cell responses has in recent years advanced from single-parameter (e.g. IFN-γ-secretion) to more complex multidimensional measurements. Numerous studies have successfully associated single-parameter functional assays of T cells with their efficacy.[1] Furthermore, it is becoming increasingly clear that *ex vivo* functional polyvalency of T cells is an important correlate of T cell efficacy.[2,3,4,5,6] Of note, it is still debated if *ex vivo* T cell polyvalency is directly [7] or indirecty [8] associated with T cell efficacy *in vivo*. Presently, no standardized or objective analytical technique exists to properly define the relationship between functional polyvalency and biological efficacy.

**Fig 1:**
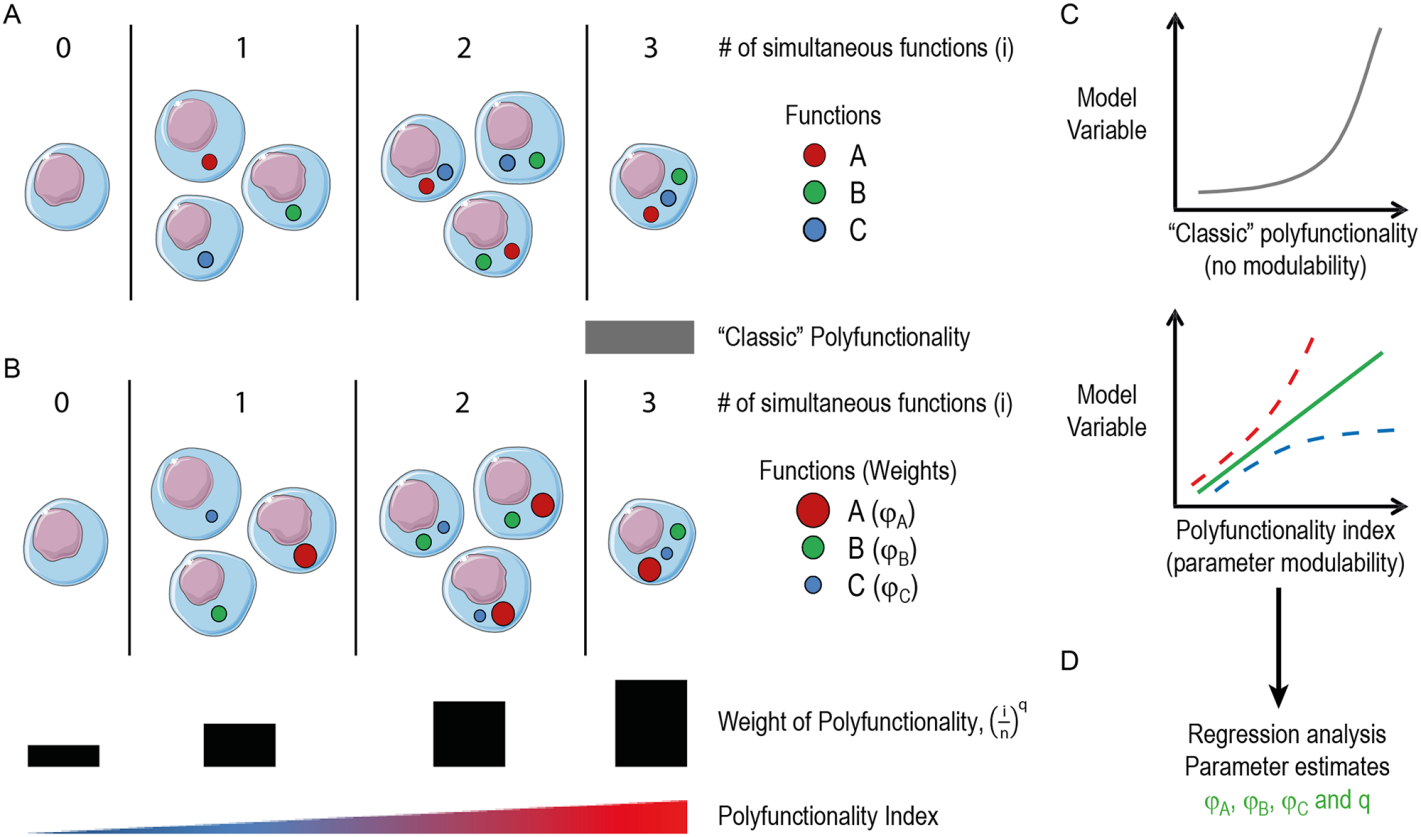
The principles of single-cell polyfunctionality analysis for modelling associated variables, such as T cell efficacy. 3 (n) bimodial effector molecules (A, B and C) were measured at the single-cell level identifying 2^3^ = 8 distinct combinatorial cell subsets, which can be stratified according to the number of simultaneous functions at the single-cell level (i). **A)** “Classical” polyfunctionality analysis generally assess only cells positive for a defined minimal number of simultaneous effector molecules (i.e. 3 simultaneous functions). **B)** Contrarily, the polyfunctionality index considers all 8 functional subsets and ingeniously parameterizes the influence of individual (φ_A_>φ_B_>φ_C_) as well as combined (*q*) functionalities. **C)** Polyfunctionality assessed with the polyfunctionality index algorithm is therefore modulable, contrary to “classic” polyfunctional analysis, enabling a more optimal fit (green line) of model variables. **D)** Using regression analysis it is feasible to obtain proper parameter estimates (φ_A_, φ_B_, φ_C_ and q), which have biological significance. Indeed, interpretation of such parameters enables an objective evaluation of the influence of individual as well as combinatorial functions on the predictive capacity of polyfunctionality with regards to a desired model variable, such as T cell efficacy.

T cell polyfunctionality, defined as the co-expression of multiple functional molecules (such as cytokines and chemokines) at the single-cell level, is measurable with high-throughput technologies, such as multiparametric flow cytometry. Multiparametric cell analysis generates very large combinatorial datasets. For instance, 3 bimodial parameters divide cells into 2^3^ = 8 so-called boolean combination subsets. In the case of T cell polyfunctionality, the complexity of such boolean datasets has been reduced to enable associations with clinical and biological parameters. Two main approaches have been applied in attempt to reduce complexity of T cell polyfunctionality, 1) polyfunctionality profiles characterized by the frequency of cells positive for 0, 1, 2, until n simultaneous parameters presented as pie diagrams (n+1 dimensions) and 2) quantitative polyfunctionality characterized by the frequency of cells that produce a defined number of functions (Fig 1A).

Polyfunctionality profiles have been instrumental for our understanding of cellular functionality, but remain difficult to interpret with appropriate statistical inference. Quantification of cellular polyfunctionality would largely facilitate statistical analysis, but so-far there is no widely accepted quantification methodology that integrates the contribution of all functional layers of T cells. Indeed, the quantitative approaches most frequently used [9,10] are restricted to the enumeration of highly polyfunctional cells and do not consider pauci-functional cells. More specifically, they do not take into account functional cells positive for a number of functions inferior to a defined lower limit and do not differentiate cells according to the number of functions for which they are positive (Fig 1A).

We have recently proposed a novel algorithm, which quantifies polyfunctionality while taking into account all functional T cells (polyfunctionality index) (Fig 1B). [11] The polyfunctionality index is easy to implement with a variety of statistical techniques, such as comparative and correlative statistics (Fig 1C). [12,13] Here, we use the algorithm to determine the importance of T cell polyfunctionality with regards to T cells capacity to kill target cells *in vitro* and control infection *in vivo*, hence T cell efficacy. We finally develop an approach with which we were able to accurately discern and grade individual functional parameters, such as effector molecules, which are important for describing the association between T cell polyfunctionality and T cell efficacy (Fig 1D). Our approach enables an objectively based exclusion of redundant functional parameters and thus reduce both cost and complexity of experimental procedures. The parameter estimates identified by our approach may serve as predictive biomarkers and identify key targets to be modulated by therapeutic interventions.

## Materials and Methods

### Selection of T cell polyfunctionality data

Contrary to the analytical approaches so-far utilized for T cell polyfunctionality analysis, the polyfunctionality index provides several components enabling an objective and quantitative assessment of the degree to which T cell efficacy is associated with T cell polyfunctionality. Moreover, we can robustly grade the impact of individual functional parameters. In two of our previous studies we showed association between T cell polyfunctionality and T cell mediated target killing [9] as well as vaccine induced control of *Leishmania major* infection [10]. We therefore selected these two datasets to more thoroughly understand the contribution of individual functional parameters with respect to T cell efficacy.

### Polyfunctional CD8^+^ T cell responses towards HIV-1

One experimental dataset was composed of T cell polyfunctionality and target killing capacity of HIV-specific CD8^+^ T cell clones analysed as previously described.[9] Briefly, T cell clones from 3 HLA B*2705 HIV-1 seropositive patients were stimulated for 6 hours with serial dilutions (10^-6^-10^-12^ M) of cognate peptide (p24 Gag KK10; residues 263–272) and analysed on a BD LSRII apparatus (BD Biosciences) for intracellular expression of IFN-γ, TNF-α, IL-2 and MIP-1β as well as surface displayed marker of recent degranulation, CD107a.

### Polyfunctional CD4^+^ T cell responses against Leishmania major

A second experimental dataset was composed of polyfunctionality profiles of *Leishmania major*-specific CD4^+^ T cells from the spleen of mice vaccinated against *Leishmania major*. These data were associated with vaccine induced protective capacity measured as the lesion size post infectious challenge as previously described.[10] Briefly, mice were vaccinated with a recombinant leishmanial polyprotein (MML) either expressed by a replication-deficient adenovirus or as protein with CpG as adjuvant. Vaccination with adenovirus was delivered once either *intramuscular* (leg) or *subcutaneous* (foot). Protein vaccination was administrated three times in intervals of two weeks. A group of mice were vaccinated with sub-lethal doses of live *Leishmania major*. T cell polyfunctionality defined by co-expression of IFN-γ, TNF-α and IL-2 was analysed by intra-cellular cytokine staining at day 28 post vaccination by *in vitro* stimulation with MML for 6 hours. Stained cells were acquired on a BD LSRII flow cytometer. Maximum lesion size was measured post intradermal challenge in both ears with metacyclic *Leishmania major* promastigotes.

### Flow cytometry data analysis

Data analysis was accomplished with FlowJo (TreeStar Inc) software. Polyfunctionality analysis was performed using Pestle and Spice softwares (Mario Roederer, ImmunoTechnology Section, VRC/NIAID/NIH)[14] as well as FunkyCells Boolean Dataminer software (www.FunkyCells.com). Primary polyfunctionality data for the two datasets analysed in the present manuscript can be found in S1 and S2 Tables.

### Ethics statement

Human blood samples were obtained following acquisition of the study participants’ written informed consent and the study protocol was reviewed and approved by local ethics committee of Pitié-Salpêtrière Hospital, Paris. This study was carried out in strict accordance with the recommendations in the Guide for the Care and Use of Laboratory Animals of The National Institutes of Health. The protocol was approved by the Vaccine Research Center Animal Care and Use Committee (protocol number VRC-04-072).

### Polyfunctionality index algorithms

In the analysis herein, we define three different, but related algorithms to represent polyfunctionality. The formulae and their application in validating their relationship with T cell efficacy are summarized in Table 1.

**Table 1:** Formulae and application of polyfunctionality algorithms in establishing their relationship with T cell efficacy.

Algorithm	Equation*	φ (Range)	PI (Range)	Modifications**	Use when…
General PI	$\frac{\overset{1}{\underset{x_{1}=0}{\sum }}\overset{1}{\underset{x_{2}=0}{\sum }}......\overset{1}{\underset{x_{n}=0}{\sum }}(1+\varphi_{\left(\begin{array}{c} x_{1}\\ x_{2}\\ :\\ x_{n} \end{array}\right)})\cdot F_{\left(\begin{array}{c} x_{1}\\ x_{2}\\ :\\ x_{n} \end{array}\right)}\cdot {\left(\frac{\overset{n}{\underset{j=0}{\sum }}x_{j}}{n}\right)}^{q}}{\left(1+\varphi_{\left(\begin{array}{c} 1\\ 1\\ :\\ 1 \end{array}\right)}\right)}$	≥ 0	[0, 1]	None	**1)** Effector molecules positively associate with T cell efficacy.**2)** Importance of effector molecules are graded according to their relevance with respect to the predicted parameter (*i*.*e*. T cell efficacy). **3)** Accounts for increasing levels of polyfunctionality
Special PI	$\overset{n}{\underset{i=0}{\sum }}F_{i}\cdot {\left(\frac{i}{n}\right)}^{q}$	NA	[0, 1]	None	**1)** Importance of effector molecules are NOT graded according to their relevance with respect to the predicted parameter (*i*.*e*. T cell efficacy). **2)** Accounts for increasing levels of polyfunctionality
Modified general PI	$\overset{1}{\underset{x_{1}=0}{\sum }}\overset{1}{\underset{x_{2}=0}{\sum }}......\overset{1}{\underset{x_{n}=0}{\sum }}(1+\varphi_{\left(\begin{array}{c} x_{1}\\ x_{2}\\ :\\ x_{n} \end{array}\right)})\cdot F_{\left(\begin{array}{c} x_{1}\\ x_{2}\\ :\\ x_{n} \end{array}\right)}\cdot {\left(\frac{\overset{n}{\underset{j=0}{\sum }}x_{j}}{n}\right)}^{q}$	(-∞, +∞) (-∞, +∞) (-∞, +∞) ≥ 0	[0, 1] [0, +∞) (-∞, +∞) [0, +∞)	None PI/PI_max_ e^PI^/(e^PI^+1) Apply general PI	**1)** Effector molecules positively, negatively, or null associate with T cell efficacy. **2)** Importance of effector molecules are graded according to their relevance with respect to the predicted parameter (*i*.*e*. T cell efficacy). **3)** Accounts for increasing levels of polyfunctionality

NA = Not Applicable, PI = Polyfunctionality Index

* For complete description of the parameters, see materials and methods section.

** These adjustments are required in the event that the PI does not range [0, 1].

#### 1. General polyfunctionality index

We previously developed the general polyfunctionality index algorithm.[11] Briefly,

$$\text{General Polyfunctionality index}=\frac{\overset{1}{\underset{x_{1}=0}{\sum }}\overset{1}{\underset{x_{2}=0}{\sum }}......\overset{1}{\underset{x_{n}=0}{\sum }}(1+\varphi_{\left(\begin{array}{c} x_{1}\\ x_{2}\\ :\\ x_{n} \end{array}\right)})\cdot F_{\left(\begin{array}{c} x_{1}\\ x_{2}\\ :\\ x_{n} \end{array}\right)}\cdot {\left(\frac{\overset{n}{\underset{j=0}{\sum }}x_{j}}{n}\right)}^{q}}{\left(1+\varphi_{\left(\begin{array}{c} 1\\ 1\\ :\\ 1 \end{array}\right)}\right)}$$ (1)

$${\text{x}}_{j}=\text{ 0 or 1}$$ (2)

$$\varphi_{\left(\begin{array}{c} x_{1}\\ x_{2}\\ :\\ x_{n} \end{array}\right)}=\left[\begin{array}{cccc} \varphi_{1} & \varphi_{2} & ... & \varphi_{n} \end{array}\right]\times \left[\begin{array}{c} x_{1}\\ x_{2}\\ :\\ x_{n} \end{array}\right]=\varphi_{1}\cdot x_{1}+\varphi_{2}\cdot x_{2}+....+\varphi_{n}\cdot x_{n}$$ (3)

$$\overset{1}{\underset{x_{1}=0}{\sum }}\overset{1}{\underset{x_{2}=0}{\sum }}......\overset{1}{\underset{x_{n}=0}{\sum }}F_{\left(\begin{array}{c} x_{1}\\ x_{2}\\ :\\ x_{n} \end{array}\right)}=1$$ (4)

$$F_{\left(\begin{array}{c} x_{1}\\ x_{2}\\ :\\ x_{n} \end{array}\right)}\geq {\text{ 0 for all x}}_{j}$$ (5)

$$\varphi_{\text{j}}\;\geq \text{0 for all }j$$ (6)

*n* > 0 is the number of functions studied. x_*j*_ indicates in a binary fashion (2) if the combinatorial T cell subset $\left(\begin{array}{c} x_{1}\\ x_{2}\\ :\\ x_{n} \end{array}\right)$ perform the j^th^ function (x_j_ = 1) or not (x_j_ = 0). $F_{\left(\begin{array}{c} x_{1}\\ x_{2}\\ :\\ x_{n} \end{array}\right)}$ is the frequency of cells performing the particular combination of functions $\left(\begin{array}{c} x_{1}\\ x_{2}\\ :\\ x_{n} \end{array}\right)$. $\left(1+\varphi_{\left(\begin{array}{c} x_{1}\\ x_{2}\\ :\\ x_{n} \end{array}\right)}\right)$ is a factor assigned to a T cell subset performing the particular combination of functions $\left(\begin{array}{c} x_{1}\\ x_{2}\\ :\\ x_{n} \end{array}\right)$ (3). *q*≥0 is the parameter that modulates the effect of increasing levels of polyfunctionality, be it in a monotonic (*q* = 1) or exponential (*q*≠1) manner, according to the number of simultaneous functions $\left(\begin{array}{c} x_{1}\\ x_{2}\\ :\\ x_{n} \end{array}\right)$ expressed by the cell subset. The algorithm requires that the sum of all $F_{\left(\begin{array}{c} x_{1}\\ x_{2}\\ :\\ x_{n} \end{array}\right)}$ equals 1 (4) and that all $F_{\left(\begin{array}{c} x_{1}\\ x_{2}\\ :\\ x_{n} \end{array}\right)}$ and all factors (*φ*_*j*_) are absolute values (5–6).

#### 2. Special polyfunctionality index

The general polyfunctionality index can be simplified to the special polyfunctionality index (7), when all effector molecules are considered to contribute equally (*φ*_*j*_ = 0 for all j).

$$\overset{n}{\underset{i=0}{\sum }}F_{i}\cdot {\left(\frac{i}{n}\right)}^{q}$$ (7)

where *F*_*i*_ is the frequency of cells performing *i* simultaneous functions.

#### 3. Modified general polyfunctionality index

Some parameterizations of the general polyfunctionality index may not be fully adapted to certain experimental conditions. The general polyfunctionality index assumes that all effector molecules contribute positively to T cell efficacy (6). Indeed, this may not be the case for anti-inflammatory and regulatory cytokines or in the case where particular cytokines define detrimental differentiation pathways. In order to account for effector molecules that have no effect or even a negative effect on T cell efficacy, we ignore assumption (6), thus letting all φ_j_ take on any value from negative to positive infinite. As a result, the denominator $\left(1+\varphi_{\left(\begin{array}{c} 1\\ 1\\ :\\ 1 \end{array}\right)}\right)$ no longer ensures that the polyfunctionality index ranges from 0–1 and was hence removed from Eq (1). Instead, the distribution of the polyfunctionality equation must be evaluated *a posteriori* based on the given inputs, in which other operations may be warranted (i.e. adjustment via dividing by the maximum PI value or inverse logit transformation—cf. Table 1). The following Eq (8) represents the modified general polyfunctionality index:

$$\overset{1}{\underset{x_{1}=0}{\sum }}\overset{1}{\underset{x_{2}=0}{\sum }}......\overset{1}{\underset{x_{n}=0}{\sum }}(1+\varphi_{\left(\begin{array}{c} x_{1}\\ x_{2}\\ :\\ x_{n} \end{array}\right)})\cdot F_{\left(\begin{array}{c} x_{1}\\ x_{2}\\ :\\ x_{n} \end{array}\right)}\cdot {\left(\frac{\overset{n}{\underset{j=0}{\sum }}x_{j}}{n}\right)}^{q}$$ (8)

### Interpretation of polyfunctionality index parameters *q* and φ

From these equations, the parameters of most interest are *q* and *φ*_*i*_ and can be estimated using appropriate regression techniques. In the instance of *q*, the values it takes are interpreted as follows:

*q* → 0: polyfunctionality is not an immune correlate.

0 < *q* < 1: polyfunctionality is a moderate immune correlate.

*q* > 1: polyfunctionality is a strong immune correlate.

In the instance of *φ*_*i*_, the values it takes on are interpreted as follows:

*φ*_*i*_ < 0: the function i contributes negatively to the polyfunctionality index.

*φ*_*i*_ = 0: the function i contributes neutrally to the polyfunctionality index.

*φ*_*i*_ > 0: the function i contributes positively to the polyfunctionality index.

Of note, the *φ*_*i*_–values are relative measures. Indeed, comparing *φ*_*i*_ with all other *φ*_*j*_ permit interpreting which functions contribute more or less to the polyfunctionality index, and therefore which functions are more relevant to measure to model or predict a given target variable.

### Modelling T cell efficacy using the special polyfunctionality index

For the experiments assessing the polyfunctionality of HIV-specific CD8^+^ T cells,[9] we employed non-linear least-squares regression with the dependent variable being percent killing and the independent variable being the resulting series expansion of the special polyfunctionality index (7), where F_i_ was determined using FunkyCells software (www.FunkyCells.com) and *i* and *n* were constants. Both the slope (β_1_) in relation to the polyfunctionality index and constant (β_0_) parameter estimates were obtained for this model. The parameter *q* was also directly calculated from this model.

A total of four separate experiments were conducted to assess the polyfunctionality of vaccine induced CD4^+^ T cell responses towards *Leishmania major*.[10] For each experiment, separate non-linear least-squares regression models were constructed using mean lesion size as the dependent variable and polyfunctionality index values from the series expansion as the independent variable (described above). In order to determine *q*, a range of values were inputted into each of the series expansions of the polyfunctionality index, while the median squared correlation coefficient (*r* ^2^) across the four regression functions was calculated for the same *q*. This resulted in a description of median *r* ^2^ as a function of *q*, from which numeric first and second derivatives were calculated using a previously defined algorithm.[15] From these, the optimal *q* was then determined by the local maximum of the concave downward function. Conveniently, this is analogous to using the referenced output option of FunkyCells Boolean Dataminer (www.FunkyCells.com) in conjunction with the Generalized Reduced Gradient non-linear iterative computation included in the Frontline Systems Solver add-in for Microsoft Excel. Graphical representation of r^2^ as a function of *q* can be accomplished using FunkyCells Track Function software (www.FunkyCells.com).

For both these methods, robustness of *q* was evaluated via bootstrapping by randomly selecting with replacement 90% of the original data (overall for experiments involving HIV-specific CD8^+^ T cell polyfunctionality and within experiments involving *Leishmania major*-specific CD4^+^ T cell polyfunctionality) and re-running the *q* computation above. 95% two-sided confidence intervals (CIs) were then constructed using the non-studentized pivotal method.

### Modelling T cell efficacy using the modified general polyfunctionality index

To assess the differential importance of individual effector molecules we employed the modified general polyfunctionality index (8). Non-linear regression models were used in a similar manner as described above, only the independent variable was the resulting series expansion of the modified general polyfunctionality index. The effector molecule-specific factor, φ_*j*_, is then estimated from the terms $\varphi_{\left(\begin{array}{c} 1\\ 1\\ :\\ 1 \end{array}\right)}$. This can be viewed as a parameter estimate of the individual slopes describing the relationship between a specific effector molecule and T cell efficacy while accounting for polyfunctionality.

### Using co-linearity to assess the redundancy of HIV-specific CD8^+^ T cell effector molecules

We first wanted to examine the probability distribution of polyfunctionality for T cells secreting a specific effector molecule. Using FunkyCells software (www.FunkyCells.com), mean frequencies of each boolean combination were calculated. These frequencies can be viewed as the conditional probability of cells secreting *i* number of effector molecules given that they secrete effector molecule *j*.

In the regression analysis, we viewed the redundancy of certain effector molecules as a co-linearity problem. In order to assess this, we used linear regression to model the frequency of cells expressing one specific effector molecule against the frequency of all other effector molecules with intercept. This resulted in the correlation coefficient, $\hat{R_{j}^{2}}$, which was then used to calculate the centered variance inflation factor (VIF) for chemokine *j*:

$${VIF}_{j}\;=\;\frac{1}{1-\hat{R_{j}^{2}}}$$

High VIF (>5) represents strong co-linearity with at least one other effector molecule and suggests that the specific cytokine can be removed from the model.

### Statistical analysis

Statistical analysis was performed using STATA (v12.1, College Station, TX, USA) and a *p*-value <0.05 was considered significant.

## Results

### T cell killing is associated with T cell polyfunctionality

We previously showed that functional avidity of 10 CD8^+^ T cell clones specific for an HLA-B27*05 restricted p24 Gag HIV epitope (KK10) was associated with polyfunctionality and HIV-suppressive activity. Indeed, CD8^+^ T cell clones of high functional avidity reacted to stimulation by antigen presenting cells loaded with KK10 at lower concentrations than CD8^+^ T cell clones of low functional avidity, both with regards to target killing and capacity to produce effector molecules separately and simultaneously.[9]

**Fig 2:**
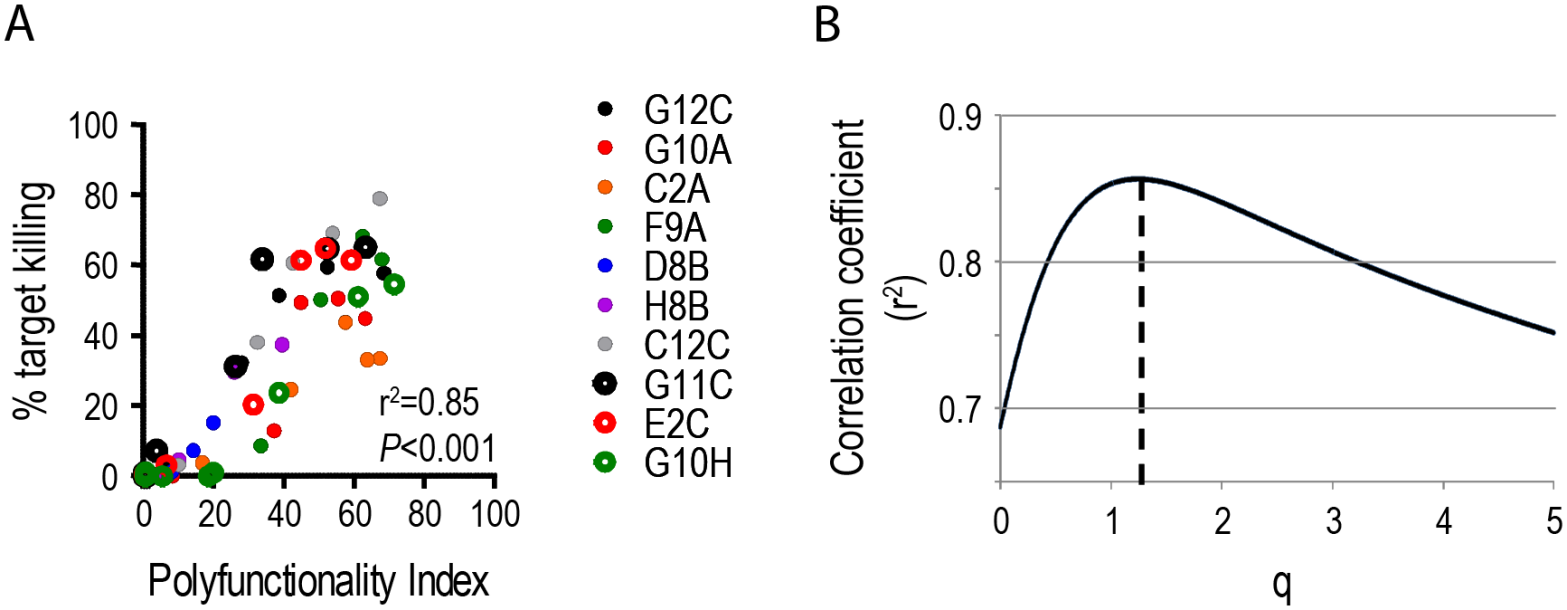
Polyfunctionality of HIV-specific CD8^+^ T cells is associated with T cell mediated target killing. *In vitro* cultured human HIV-specific (KK10) CD8^+^ T cell clones were incubated with KK10-loaded target cells (HLA-B*2705^+^ lymphoblastoid cell lines). To measure T cell killing a standard chromium release assay was employed using ^51^Cr charged target cells loaded with a serial dilution of KK10-antigen [10^-6^M—10^-13^M]. Non-^51^Cr charged target cells with an identical serial dilution of KK10-antigen were employed to analyse T cell polyfunctionality by multiparametric flow cytometry (CD107a, IFN-γ, TNF-α, IL-2, MIP-1β). **A)** Scatter plots showing the association between T cell killing (^51^Cr release / max ^51^Cr release) and T cell polyfunctionality quantified as the polyfunctionality index (*q* = 1). **B)** Non-linear regression for all T cell clones identifies the optimal q-value as 1.24.

To further understand the relationship between polyfunctionality and T cell efficacy, we employed the polyfunctionality index. In the first approach, we used the special polyfunctionality index, which enables an estimation of the impact of polyfunctionality, in the special condition where all effector molecules are considered equally important (cf. materials and methods section). Briefly, target killing was associated with polyfunctionality index values of individual T cell clones at different target concentrations. In the initial model, *q* was equal to 1, thereby reflecting a situation where the number of simultaneous effector molecules produced by a T cell adds to the polyfunctionality index in a monotonic manner. We observed a strong correlation between polyfunctionality and target killing (Fig 2A, r^2^ = 0.85, *P*<0.001) with a strong positive slope of 0.87 (95%CI: [0.80;0.95], *p*<0.001). The correlation coefficient changes with varying values of *q* (Fig 2B). Using least-squares regression, we then estimated which *q* resulted in the optimal fit, obtaining a value of *q* = 1.24 with a correlation coefficient of 0.86 (Table 2). The capacity of T cell polyfunctionality to predict T cell efficacy as measured by target killing is quantified by the parameter *q*. The fact that *q*>0 shows that polyfunctionality is a correlate of T cell efficacy, which contributes monotonically (*q* = 1) or slightly exponentially (*q*>1) to the predictive capacity of T cell polyfunctionality. Of note, for *q*→0 the polyfunctionality index defines the frequency of cells producing at least one effector molecule. Our model therefore allows a direct statistical comparison between the predictive capacity of functional T cell quality (q>0) and quantity (q→0).

**Table 2:** Linear regression model of CD8^+^ T cell mediated target killing as a function of polyfunctionality based on five effector molecules.

Parameters		Model 1		Model 2	
		Estimate (95% CI)	*p*	Estimate (95% CI)	*p*
		1.24 (0.85, 1.63)	**<0.001**	3.05 (1.30, 4.81)	**0.001**
*φ*					
	CD107a	—		-0.96 (-3.96, 2.03)	0.5
	TNF-α	—		-5.65 (-12.38, 1.08)	0.10
	IFN-γ	—		1.04 (-0.36, 2.44)	0.14
	MIP-1β	—		5.86 (-4.32, 16.03)	0.3
	IL-2	—		-0.62 (-1.62, 0.39)	0.2
Correlation (R^2^)		0.8563		0.9144	

### Control of Leishmania major infection is associated with T cell polyfunctionality

Whereas the above dataset is based on robust and elaborate *in vitro* analysis of human T cell functionality, we wanted to investigate whether quantitative assessment of T cell polyfunctionality represents a marker of *in vivo* T cell efficacy. We previously reported that polyfunctionality of vaccine induced *Leishmania major*-specific CD4^+^ T cells were strongly correlated with the protective capacity against an infectious challenge with live *Leishmania major*.[10] Indeed, the size of the skin lesion induced by the *Leishmania major* infection was inversely correlated with the frequency of antigen-specific CD4^+^ T cells producing IFN-γ, TNF-α and IL-2, simultaneously.

**Fig 3:**
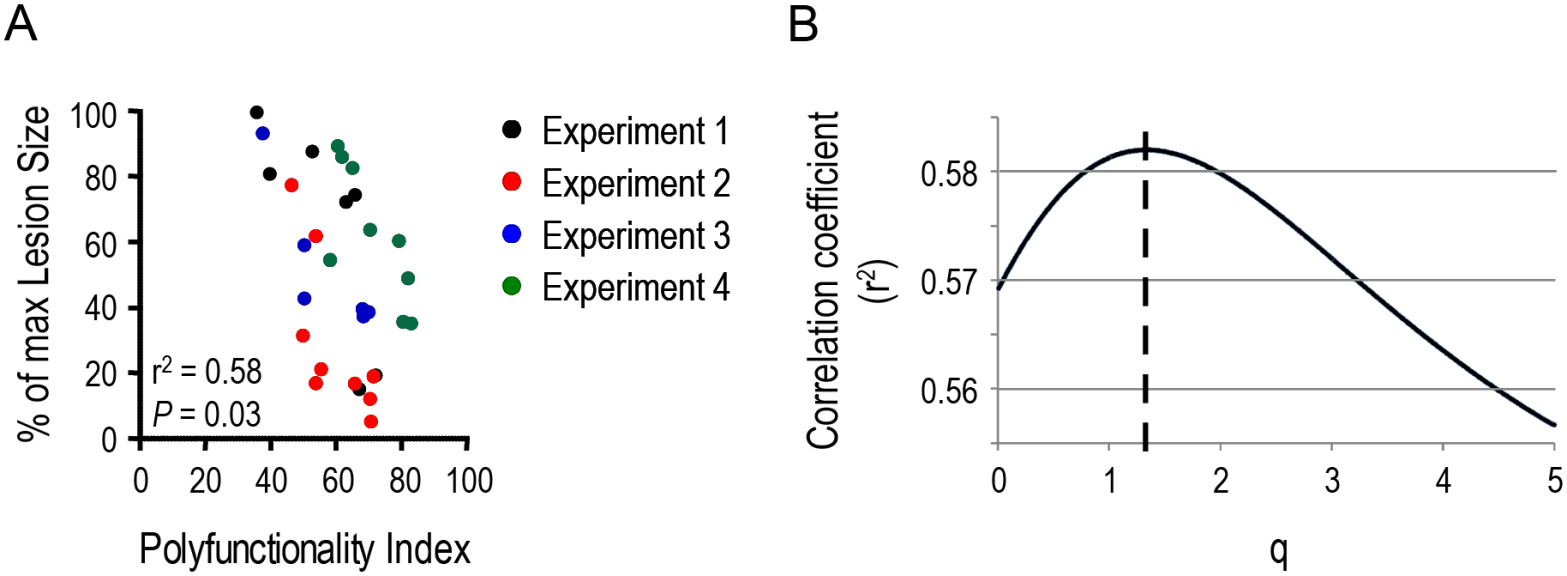
Polyfunctionality of *Leishmania major*-specific CD4^+^ T cells is associated with the degree of protection induced by vaccination. Mice were vaccinated with *Leishmania major* antigens employing different antigen preparations (recombinant leishmanial polyprotein (MML) with adjuvant (CpG) and replication-defective adenovirus expressing MML), different doses and different routes of injection. Mice were challenged 28 days post vaccination by intradermal ear injections of *Leishmania major* carrying parasites. *Leishmania major* protection levels were measured as peak lesion size. Spleen derived lymphocytes harvested 28 days post vaccination were stimulated with anti-CD28 and MML for 6 hours and analysed for the production of IFN-γ, TNF-α and IL-2 by multiparametric flow cytometry. Experiments were repeated in four independent experiments **A)** Scatter plots show the association between normalized lesion size and the T cell polyfunctionality quantified as the polyfunctionality index (*q* = 1). **B)** Non-linear regression analysis over the 4 experiments identified the optimal *q*-value as 1.33. Statistical analysis was conducted with parametric aggregate correlation statistics.

Employing the same methodology as outlined for the *in vitro* generated dataset on T cell killing above, we used the polyfunctionality index to further understand the relationship between T cell polyfunctionality and protection against *Leishmania major* infection. As above, we used the special polyfunctionality index considering all effector molecules equally important (cf. materials and methods section). We first regressed polyfunctionality index values for *Leishmania major*-specific CD4^+^ T cells up-against resistance to *Leishmania major* infection (peak skin lesion size post challenge) for *q* = 1. We observed a strong negative slope of -5.29 (95%CI: [-6.26; -4.27], p<0.001) producing a correlation coefficient of 0.58 (*P* = 0.025) between polyfunctionality and *Leishmania major* induced skin lesion size (average statistics for 4 independent experiments—Fig 3A). The correlation coefficient changes with varying levels of *q* (Fig 3B). Using least-squares regression analysis as outlined above, the optimal *q*-value with the highest median correlation coefficient was *q* = 1.33. The robustness of the optimal *q*-value was analysed by bootstrapping (cf. materials and methods), giving an optimal *q* = 1.9 and a 95% confidence interval of [-2.04:5.84]. Interestingly, the Leishmania major-specific response size defined as the frequency of CD4+ T cells producing cytokines upon stimulation with Leishmania major, is less well correlated with protection as measured by lesion size (S1A Fig, r^2^ = 0.36, *P* = 0.12). Moreover, response size and polyfunctionality of the Leishmania major-specific T cell response do not correlate (S1B Fig, r^2^ = 0.15, *P* = 0.48). The capacity of CD4^+^ T cell polyfunctionality to predict vaccine efficacy, as measured by the resistance evoked towards *Leishmania major* infection, was quantified by the parameter *q*, and demonstrates that antigen-specific CD4^+^ T cell polyfunctionality is a superior correlate of vaccine efficacy compared with response size.

### Differential evaluation of effector molecules does not increase the predictive power of T cell efficacy

Our analysis demonstrates that polyfunctionality is an important positive immune correlate for 1) T cell mediated killing of HIV infected target cells and 2) *in vivo* control of *Leishmania major* infection. We subsequently wanted to elucidate if certain effector functions would be more important for the correlates than others. We therefore performed a least-squares regression analysis of T cell efficacy as a function of polyfunctionality, defined by the polyfunctionality index.

For T cell efficacy measured as target killing, the analysis was performed considering all effector molecules equally important (model 1—special polyfunctionality index as above) and by including effector molecules as parameter estimates (model 2—modified general polyfunctionality index). The two analysis were able to model 85.63% (model 1) and 91.44% (model 2) of the model variability (Table 2). The parameter estimates showed that *q* was significantly larger than 0 for model 1 (*q* = 1.24; 95% CI: 0.85–1.63, *p*<0.0001). In concordance with model 1 we performed a second analysis taking into account the importance of individual effector molecules (model 2), which found *q* to be significantly larger than 1 (*q* = 3.05; 95% CI: 1.30–4.81, *p* = 0.02) (Fig 2B and Table 2). None of the φ-values associated with the 5 effector molecules, CD107a, IFN-γ, IL-2, MIP-1β and TNF-α, respectively, were significantly different from 0 (Model 2, Table 2). In other words, polyfunctionality is the primary predictor of T cell efficacy and all measured effector molecules contribute equally to the predictive capacity of the polyfunctionality index with regards to T cell efficacy.

We attempted a similar analysis for the *Leishmania major* vaccine study. However, the variability of this dataset, did not allow the polyfunctionality index parameters to converge. We performed a power analysis using fixed parameters from previous models (S2 Fig), demonstrating that 75 groups of mice would be needed to achieve 80% power in order to determine a significant difference for a true φ_i_ = 1. The inherent data variability is most likely due to the *in vivo* nature of the mouse experiments. A larger study is required to reduce some of this variability, thus allowing full evaluation on whether certain effector molecules contribute more to the predictive capacity of polyfunctionality with regards to vaccine protection.

### Information redundancy between effector molecules

From our previous studies it is clear that effector molecules expressed as a result of cellular stimulation, such as antigen-specific stimulation, appear in quantities ranked according to effector molecules but increasing with the intensity of stimulation in a synchronous manner.[9] Cellular pathways activated by antigen-specific stimulation and leading to expression of effector molecules have been shown to include a significant amount of redundancy.[16] We therefore wanted to evaluate if the effector molecules measured in our experimental dataset would be redundant in a polyfunctional setting.

**Fig 4:**
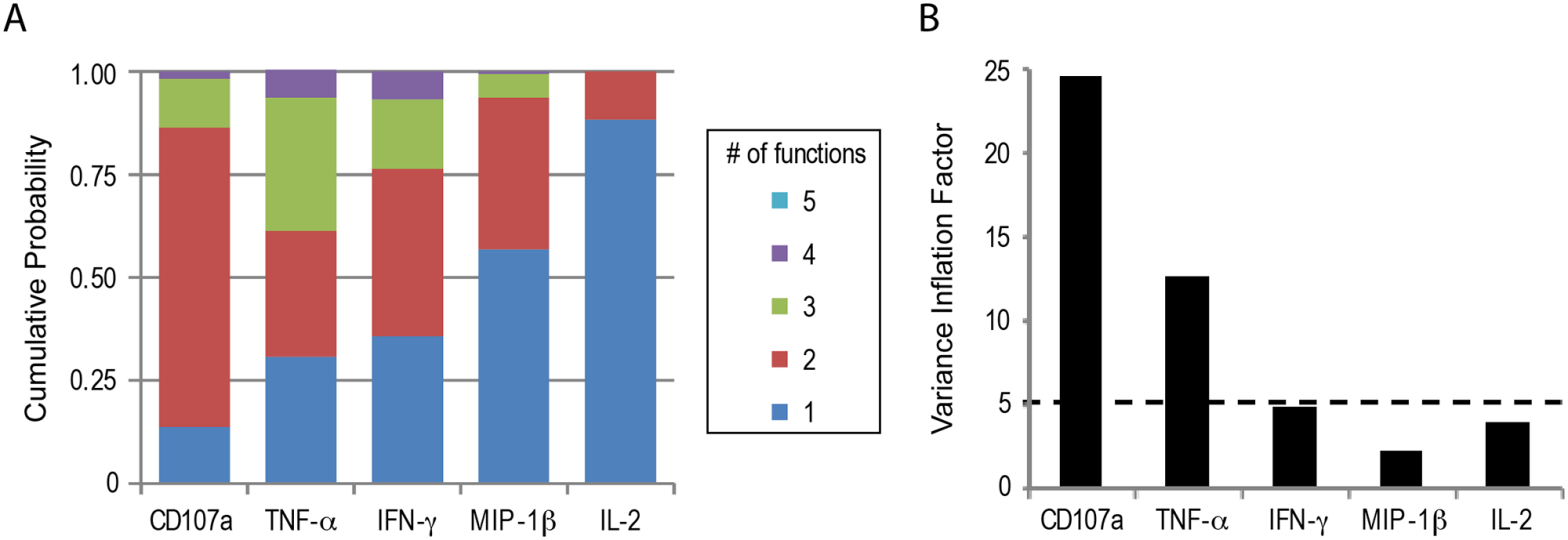
Redundancy of effector molecules with regards to the association between HIV-specific CD8^+^ T cell polyfunctionality and T cell mediated target killing. *In vitro* cultured human HIV-specific (KK10) CD8^+^ T cell clones were incubated with a serial dilution of KK10-antigen [10^-6^M—10^-13^M] loaded target cells. T cell polyfunctionality was analysed by multiparametric flow cytometry (CD107a, IFN-γ, TNF-α, IL-2, MIP-1β). **A)** Stacked bar diagram indicates the probability distribution of T cells expressing 5, 4, 3, 2 or 1 simultaneous effector molecules, given that they express one particular effector molecule. **B)** Variance Inflation Factor (VIF) analysis of the 5 effector molecules shows the lowest VIF for each molecule after iterative retraction of the effector molecule with the highest VIF>5 (cf. Table 3).

We first analysed the probability of expressing 1, 2, until n simultaneous effector molecules for cells expressing a given effector molecule. This analysis was performed for all effector molecules within our datasets. For the dataset charting polyfunctionality of HIV-specific CD8^+^ T cell clones; CD107a, IFN-γ and TNF-α have very similar probability distributions, secreting equally zero, one or two other effector molecules. On the contrary, MIP-1β was mostly secreted alone or with another effector function, while IL-2 was almost always secreted by itself (Fig 4A). This analysis suggests that there might be redundancy between CD107a, IFN-γ and TNF-α. Consequently, we analysed the variance inflation factors (VIF) for each effector molecule based on the modelling of target killing (Table 3 and Fig 4B). This analysis (model 1) showed that CD107a presents substantial co-linearity with at least one other effector molecule (VIF_CD107a_ = 24.62). We consequently removed CD107a from the model and reanalysed the VIF associated with the remaining effector molecules (model 2). In model 2, TNF-α continued to be highly correlated with at least one other effector molecule (VIF_TNF-α_ = 12.63). We therefore also removed TNF-α from the model (model 3). In this final model, the VIFs of IFN-γ, IL-2 and MIP-1β were all low (VIF<5), indicating that the remaining three cytokines are non-redundant. (Table 3 and Fig 4B)

**Table 3:** Variance inflation factors (VIF) of 5, 4 and 3 (model 1, 2 and 3, respectively) HIV-specific CD8^+^ T cell effector molecules.

Parameters	VIF		
	Model 1	Model 2	Model 3
CD107a	24.62	-	-
TNF-α	21.42	12.63	-
IFN-γ	9.75	9.35	4.81
MIP-1β	6.91	4.69	3.82
IL-2	2.53	2.52	2.20

Using this information, we finally performed a non-linear regression model of target killing as a function of T cell polyfunctionality including the three non-redundant effector molecules (IFN-γ, IL-2 and MIP-1β) using the modified general polyfunctionality equation. The methodology was identical to the one applied to all 5 effector molecules above. The result confirmed the previous analysis, as none of the 3 effector molecules had *φ*-values significantly different from 0 (Table 4). Of note, in concordance with the VIF analysis, this model of target killing explained essentially the same amount of variability as the model including 5 effector molecules (88.99% versus 91.44%). Altogether, we demonstrate a robust methodology to identify redundant functional markers, which can consequently be excluded in future studies based on similar biological and clinical settings.

**Table 4:** Linear regression model of CD8^+^ T cell mediated target killing as a function of polyfunctionality based on three variance-optimal effector molecules.

Parameters		Model 1		Model 2	
q	φ	Estimate (95% CI)	*p*	Estimate (95% CI)	*p*
		1.26 (1.07, 1.45)	**<0.001**	3.93 (0.83, 7.04)	**0.01**
	IFN-γ	—		-6.07 (-30.40, 18.27)	0.6
	MIP-1β	—		-3.32 (-8.38, 1.73)	0.2
	IL-2	—		8.79 (-20.26, 37.84)	0.6
Correlation (R^2^)		0.8473		0.8899	

## Discussion

Our study demonstrates the capacity of the polyfunctionality index to objectively quantify to which degree T cell polyfunctionality should be considered an immune correlate of T cell efficacy. We show that T cell polyfunctionality is indeed a superior correlate of T cell efficacy both *in vitro* and *in vivo* as compared with T cell response size. In one biological setting we analysed the efficacy of HIV-specific CD8^+^ T cell clones to kill target-cells *in vitro*. In a second biological setting we analysed the efficacy of vaccine induced *Leishmania major*-specific CD4^+^ T cells to control *Leishmania major* infection *in vivo*. For both studies we demonstrate that our prediction model of T cell efficacy considers the number of simultaneously expressed effector molecules increasingly more informative. Of note, the importance of polyfunctionality with regards to both HIV-specific CD8^+^ and *Leishmania major*-specific CD4^+^ T cell efficacy is very similar, despite the two otherwise unrelated biological settings. This proposes that general trends across clinical and biological settings may be found, although this hypothesis will take further studies to verify.

Oftentimes, experimental assessment of T cell efficacy requires procedures that are technically challenging and/or non-ethical. E.g. human vaccination trials most often can only await clinical infectious events as it would be unethical to perform infectious challenge experiments on vaccinated subjects. In the same line, pre-clinical vaccination protocols in animals may not have a clinically relevant infectious model (e.g. SIV versus HIV infection). In the latter cases it may be necessary to create *in vitro* experimental procedures to assess T cell efficacy. It would therefore be of outmost importance to define robust immune correlates of T cell efficacy based on assays that are feasible to perform in terms of ethics, cost and standardization. Detection of a battery of effector molecules secreted by antigen-specific T cells fulfils these criteria, as it only requires a blood sample and a fairly labour non-intensive experimental procedure. From the inputs obtained in the study herein, the polyfunctionality index can be used to establish a comprehensive score for T cell activity, which, from our data, is a robust predictor of T cell efficacy. Of note, the polyfunctionality index is calculated from the frequency of all combinations of effector molecules, but does not presently integrate the quantity of effector molecules (accessible as signal intensity, e.g. fluorescent intensity). Our previous works demonstrate that the quantity of effector molecules defines a characteristic hall mark of CD4 Th-type subsets [17] and is associated with vaccine efficacy [10]. It might therefore be possible to further improve the polyfunctionality index by integrating the quantity of effector molecules. Future studies aiming to examine T cell efficacy among subpopulations of HIV-1 or *Leishmania major* infected individuals are facilitated by polyfunctionality as a proxy.

However, there is one major caveat for using polyfunctionality in that each infection and set of effector molecules must be associated with a clearly definable marker of T cell efficacy. This is all the more important as the impact of T cell polyfunctionality in various biological and clinical settings could be heterogeneous. Indeed, for HIV-specific CD8^+^ T cells we associated two very closely related measures, T cell killing and polyfunctionality of T cells presented to the same target in the same type of *in vitro* assay. Conversely, measuring the efficacy of *Leishmania major*-specific CD4^+^ T cells as the capacity of a mouse to control *Leishmania major* infection is more biologically distant to the T cell polyfunctionality assay. Noteworthy, our results observed for datasets derived from these two biologically and clinically distinct phenomena are very homogeneous. To know if our observations are widely applicable, the outlined approach should be applied in more studies associating T cell polyfunctionality with T cell efficacy. Single-cell polyfunctionality is measurable by multiple techniques for many cell types and many different panels of effector molecules.[9,13,18] The approach outlined in the present work is applicable to all types of single-cell polyfunctionality measures. Our study defines a robust methodology to identify the most important parameters (polyfunctionality and/or effector molecules) contributing to a given polyfunctionality analysis. We therefore believe that past and future studies including single-cell polyfunctionality measures could readily benefit from our analytical approach to increase biological insight.

Two important aspects of our work are to 1) identify potential redundancy between effector molecules and 2) define if non-redundant effector molecules contribute differentially or equally to the immune correlate defined by the polyfunctionality index.

To answer the first question we performed a variance inflation factor analysis of our datasets. For the five effector molecules analysed in our *in vitro* CD8^+^ T cell assay, we successfully identified three non-redundant effector molecules (IFN-γ, MIP-1β and IL-2). To define their contribution to the polyfunctionality immune correlate, we employed the modified general polyfunctionality index. In concordance with the variance inflation factor analysis this model demonstrated similar model fit compared to the model with five effector functions. None of the three effector molecules measured in the *Leishmania major* vaccination dataset were redundant. Of note, redundancy is here uniquely linked to our predictive model and by no means linked with the biological roles of the monitored effector molecules.

The second question was answered using regression model analysis. The regression models established for the two experimental settings presented here demonstrate that polyfunctionality (*q*) is an important immune correlate of T cell efficacy. Of note, our mathematical model permits the identification of the better immune correlate of T cell efficacy between polyfunctionality and response size. Response size is here defined as functional T cells irrespective of the number of simultaneously produced effector molecules. Our *in vitro* and *in vivo* data unanimously demonstrate that polyfunctionality is a superior immune correlate as compared with response size. From the HIV-specific target killing dataset we fully delineated all parameter estimates including estimates of the impact of individual effector molecules (*φ*-values). Of note, no effector molecule impacted the polyfunctionality index immune correlate significantly different compared to other effector molecules. We recognize that the *in vitro* nature of our dataset could hampen the effect of certain effector molecules, such as chemo-attractants, whose effect would be better represented in *in vivo* studies. A recent theoretic study performed kinetic mathematical modelling of HIV pathogenicity, studying among several other parameters HIV-specific CD8^+^ T cell polyfunctionality. Of note, they demonstrate that T cell efficacy measured as the control of viral burden is correlated with T cell polyfunctionality.[19] Moreover, in concordance with our findings they demonstrate that T cell efficacy is mediated by the simultaneous production of effector molecules with no single effector molecule being more responsible than others.

Another recent study dissected the efficacy of CD8^+^ T cells in protecting against pulmonary *Yersinia pestis* infection, showing that TNF-α and IFN-γ effector molecules were crucial for *in vivo* protection, whereas perforin-driven cytotoxicity was not.[7] The previous study elegantly demonstrated the feasibility and importance of identifying the effector molecules contributing to T cell efficacy, however their use of mouse knock-out models to discern important versus non-important effector molecules is laborious and non-applicable to human studies. Our study provides an alternative, generalizable, non-laborious and low-cost methodology to discern the ranking of effector molecules according to their contribution to T cell efficacy both for animal and more importantly human studies.

In conclusion, we propose a methodology to identify robust parameter estimates rendering the polyfunctionality index an immune correlate of T cell efficacy. Of note, the parameter estimates has an associated biological interpretation, which estimates the importance of individual as well as combined effector molecule expression at the single-cell level. Our data demonstrate that polyfunctionality represents a significantly more effective immune correlate of T cell efficacy compared with individual effector functions. Furthermore, the approach outlined here also allows an identification of non-redundant effector molecules, which will allow a more cost-effective T cell analysis and thus improve research and future health care budgets.

## Supporting Information

S1 Fig *Percentage of Leishmania major*-reactive CD4^+^ T cells (response size) is only weakly associated with the degree of protection induced by vaccination and non-associated with their polyfunctionality. Mice were vaccinated with *Leishmania major* antigens employing different antigen preparations (recombinant leishmanial polyprotein (MML) with adjuvant (CpG) and replication-defective adenovirus expressing MML), different doses and different routes of injection. Mice were challenged 28 days post vaccination by intradermal ear injections of *Leishmania major* carrying parasites. *Leishmania major* protection levels were measured as peak lesion size. Spleen derived lymphocytes harvested 28 days post vaccination were stimulated with anti-CD28 and MML for 6 hours and analysed for the production of IFN-γ, TNF-α and IL-2 by multiparametric flow cytometry. Experiments were repeated in four independent experiments **A)** Scatter plots show the association between normalized lesion size and *Leishmania major*-specific response size quantified as the frequency of CD4^+^ T cells producing cytokines upon stimulation with *Leishmania major* antigens. **B)** Scatter plots show the association between *Leishmania major*-specific CD4^+^ T cell polyfunctionality quantified as the polyfunctionality index (*q* = 1.33) and *Leishmania major*-specific response size. Statistical analysis was conducted with parametric aggregate correlation statistics.
(TIF)

S2 Fig Power analysis identifying the minimum number of experimental units allowing the identification of *φ*_1_. Power (1-β) analysis involving the properties of *φ* are depicted. The first set of simulations tested whether φ_1_ was significantly different from 0, given that the true value of φ_1_ is 1. Using the same simulated conditions, but only modifying the true values of the other φ’s, there appears to be no influence on power (all solid markers). When φ_1_ is increased two-fold, we see that power increases substantially at all levels of N (all hollow markers). Experimental units (N) in this case are groups of mice (each group conposed of 3–4 mice) under the same experimental conditions, since these groups were within-averaged during power analysis.
(TIF)

S1 Table Dynamic table with raw polyfunctionality data for HIV-specific CD8+ T cell clones and corresponding target killing capacity. The dynamic table automatically calculate polyfunctionality index values for each sample upon modification of q- and *φ*-values. Further explanation can be found in the abbreviation datasheet included.
(XLSX)

S2 Table Dynamic table with raw polyfunctionality data for Leishmania major-specific CD4+ T cells and corresponding protection levels measured as peak lesion size post challenge. The dynamic table automatically calculate polyfunctionality index values for each sample upon modification of q- and *φ*-values. Further explanation can be found in the abbreviation datasheet included.
(XLSX)
