# Correction: Pathogen-Specific T Cell Polyfunctionality Is a Correlate of T Cell Efficacy and Immune Protection

**DOI:** 10.1371/journal.pone.0138395

**Published:** 2015-09-14

**Authors:** Anders Boyd, Jorge R. Almeida, Patricia A. Darrah, Delphine Sauce, Robert A. Seder, Victor Appay, Guy Gorochov, Martin Larsen

The following information is missing from the Competing Interests section: The authors have read the journal's policy and have the following conflicts: M.L., D.S. and G.G. are inventors of the polyfunctionality index (patent number: WO2013127904) and M.L. is proprietary owner of the Funky Cells ToolBox software (www.FunkyCells.com). This does not alter adherence to all the PLOS ONE policies on sharing data and materials.

## References

[1] Boyd A, Almeida JR, Darrah PA, Sauce D, Seder RA, Appay V, et al (2015) Pathogen-Specific T Cell Polyfunctionality Is a Correlate of T Cell Efficacy and Immune Protection. PLoS ONE 10(6): e0128714 doi: 10.1371/journal.pone.0128714 26046523 10.1371/journal.pone.0128714 PMC4457486

