# Supplementary figures and images for: Pathogen-Specific T Cell Polyfunctionality Is a Correlate of T Cell Efficacy and Immune Protection
Source: PLoS One. 2015 Jun 5;10(6):e0128714. doi: 10.1371/journal.pone.0128714 (PMC4457486; doi:10.1371/journal.pone.0128714)

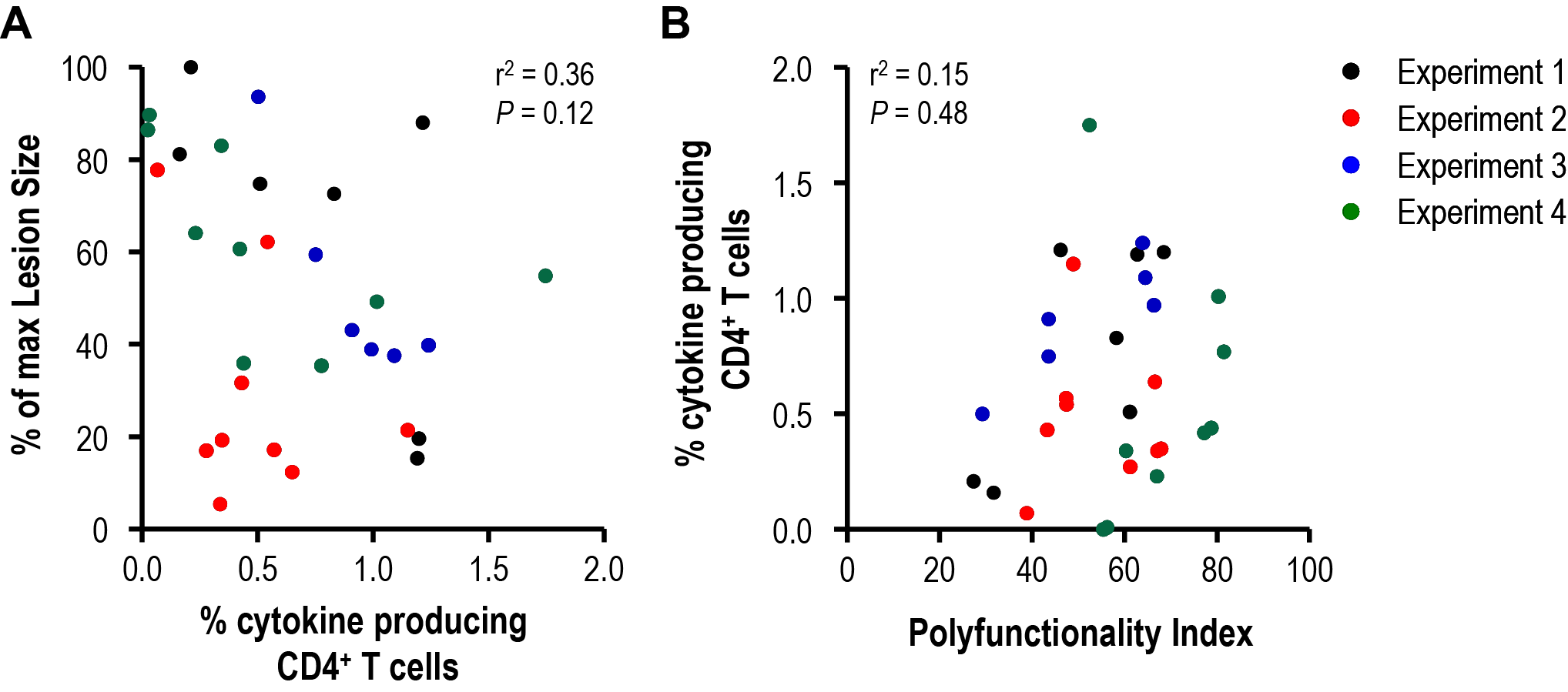

Supplement: S1 Fig — Mice were vaccinated with Leishmania major antigens employing different antigen preparations (recombinant leishmanial polyprotein (MML) with adjuvant (CpG) and replication-defective adenovirus expressing MML), different doses and different routes of injection. Mice were challenged 28 days post vaccination by intradermal ear injections of Leishmania major carrying parasites. Leishmania major protection levels were measured as peak lesion size. Spleen derived lymphocytes harvested 28 days post vaccination were stimulated with anti-CD28 and MML for 6 hours and analysed for the production of IFN-γ, TNF-α and IL-2 by multiparametric flow cytometry. Experiments were repeated in four independent experiments A) Scatter plots show the association between normalized lesion size and Leishmania major-specific response size quantified as the frequency of CD4+ T cells producing cytokines upon stimulation with Leishmania major antigens. B) Scatter plots show the association between Leishmania major-specific CD4+ T cell polyfunctionality quantified as the polyfunctionality index (q = 1.33) and Leishmania major-specific response size. Statistical analysis was conducted with parametric aggregate correlation statistics. (TIF) [file pone.0128714.s001.tif]

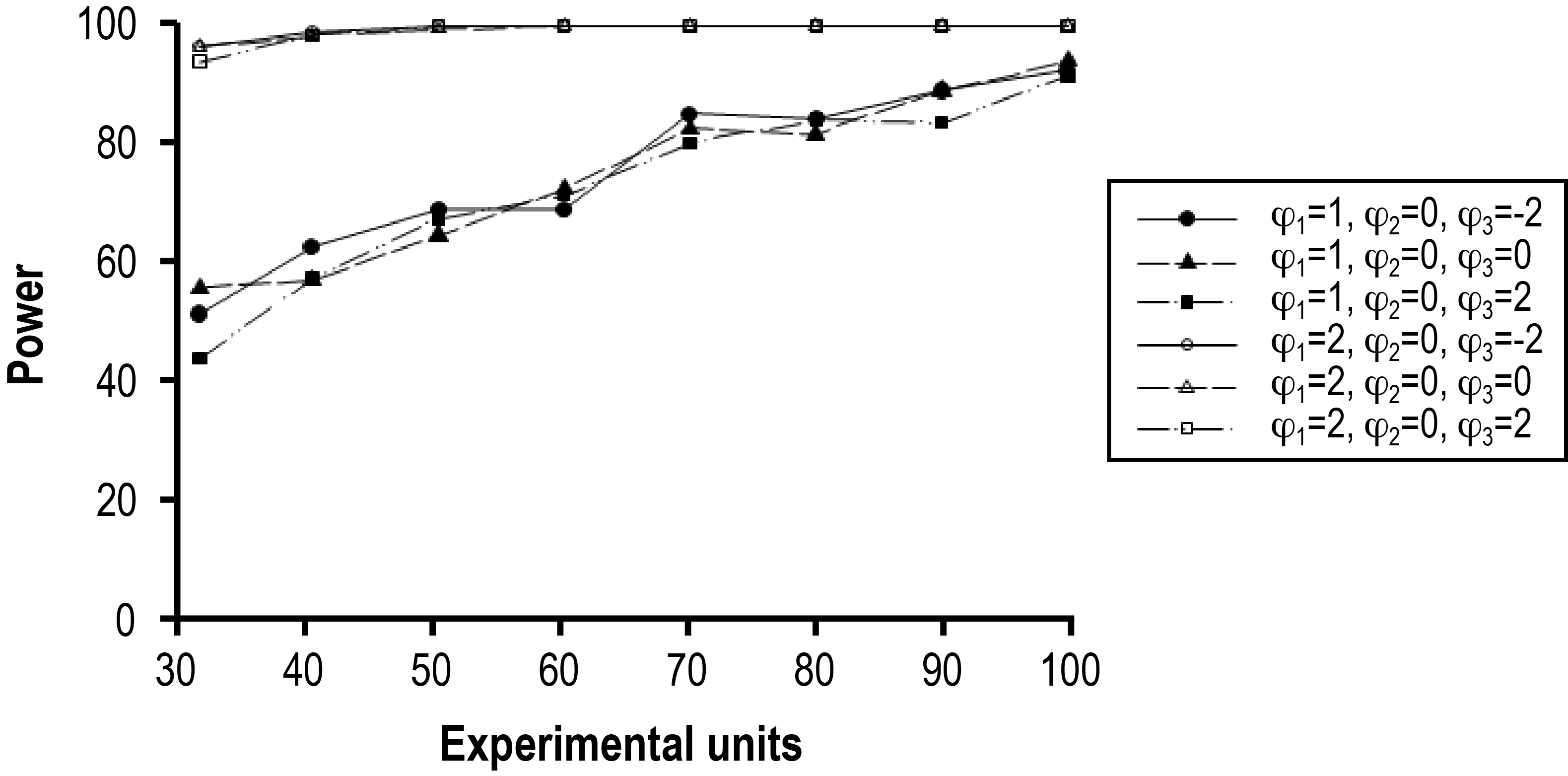

Supplement: S2 Fig — Power (1-β) analysis involving the properties of φ are depicted. The first set of simulations tested whether φ1 was significantly different from 0, given that the true value of φ1 is 1. Using the same simulated conditions, but only modifying the true values of the other φ’s, there appears to be no influence on power (all solid markers). When φ1 is increased two-fold, we see that power increases substantially at all levels of N (all hollow markers). Experimental units (N) in this case are groups of mice (each group conposed of 3–4 mice) under the same experimental conditions, since these groups were within-averaged during power analysis. (TIF) [file pone.0128714.s002.tif]
